# Exploring potential of vaginal *Lactobacillus* isolates from South African women for enhancing treatment for bacterial vaginosis

**DOI:** 10.1371/journal.ppat.1008559

**Published:** 2020-06-04

**Authors:** Anna-Ursula Happel, Brian Kullin, Hoyam Gamieldien, Nicole Wentzel, Chambrez Z. Zauchenberger, Heather B. Jaspan, Smritee Dabee, Shaun L. Barnabas, Shameem Z. Jaumdally, Janan Dietrich, Glenda Gray, Linda-Gail Bekker, Remy Froissart, Jo-Ann S. Passmore

**Affiliations:** 1 Department of Pathology, Institute of Infectious Disease and Molecular Medicine (IDM), University of Cape Town, Cape Town, South Africa; 2 Seattle Children’s Hospital, Seattle, United States of America; 3 Family Centre for Research with Ubuntu (FAMCRU), Stellenbosch University, Tygerberg, South Africa; 4 Perinatal HIV Research Unit (PHRU), Faculty of Health Sciences, University of the Witwatersrand, Johannesburg, South Africa; 5 Health Systems Research Unit, South African Medical Research Council, Cape Town, South Africa; 6 Desmond Tutu HIV Foundation, University of Cape Town, Cape Town, South Africa; 7 UMR MIVEGEC CNRS-IRD-UM, University Montpellier, Montpellier, France; 8 NRF-DST CAPRISA Centre of Excellence in HIV Prevention, Cape Town, South Africa; 9 National Health Laboratory Service (NHLS), Cape Town, South Africa; National Institutes of Health; National Cancer Institute, UNITED STATES

## Abstract

Antibiotics continue to be the standard-of-care for bacterial vaginosis (BV), although recurrence rates are high. Vaginal probiotics may improve durability of BV treatment, although few probiotics for vaginal health contain *Lactobacillus* spp. that commonly colonize the lower female genital tract. Characteristics of vaginal *Lactobacillus* strains from South African women were evaluated for their probiotic potential *in vitro* compared to strains from commercial vaginal products, including growth at varying pHs, ability to lower pH, produce D-/L-lactate and H_2_O_2_, influence growth of BV-associated *Gardnerella vaginalis* and *Prevotella bivia*, adherence to cervical cells and susceptibility to antibiotics. Fifty-seven *Lactobacillus* strains were purified from cervico-vaginal fluid, including *L*. *crispatus*, *L*. *jensenii*, *L*. *gasseri*, *L*. *mucosae*, and *L*. *vaginalis*. *L crispatus* strains grew better at pHs below 4.5 and lowered pH more effectively than other strains. Production of D-/L-lactate and H_2_O_2_ varied between *Lactobacillus* species and strains. *Lactobacillus* strains generally inhibited *P*. *bivia* more uniformly than *G*. *vaginalis* isolates. All vaginal *Lactobacillus* isolates were resistant to metronidazole while susceptibility to clindamycin varied. Furthermore, vaginal *Lactobacillus* strains tended to be broadly susceptible to penicillin, amoxicillin, rifampicin and rifabutin. Whole-genome-sequencing of five of the best-performing vaginal *Lactobacillus* strains confirmed their likely safety, due to antimicrobial resistance elements being largely absent, while putative intact prophages were present in the genomes of two of the five strains. Overall, vaginal *Lactobacillus* strains largely performed better in these *in vitro* assays than probiotic strains currently used in probiotics for vaginal health. Including the best-performing vaginal *Lactobacillus* isolates in a region-specific probiotic for vaginal health may result in improved BV treatment options.

## Author summary

*Lactobacillus* species in the lower reproductive tract of healthy women lower vaginal pH and protect against sexually transmitted infections. However, women commonly suffer from bacterial vaginosis, a disruption in the optimal *Lactobacillus*-dominated genital microbiota to a more diverse one marked by higher pH, vaginal discharge and inflammation. Bacterial vaginosis is associated with adverse pregnancy outcomes and increased risk of sexually transmitted infections, including HIV. While antibiotics are the standard-of-care for bacterial vaginosis, most cases recur within six-months. Probiotics that include *lactobacilli* have been explored to improve treatment, although the majority of products do not contain species commonly found in the vagina. Here, we characterised a large panel of vaginal *Lactobacillus* strains from young African women (including growth, adhesion to host cells, ability to produce antimicrobial products, and pathogen inhibition), compared to isolates from commercial probiotic products for vaginal health. We also sequenced the genomes of top-performing isolates. Several vaginal *Lactobacillus* strains exhibited better probiotic profiles than commercial strains, suggesting that they would be beneficial in the development of probiotic treatment of bacterial vaginosis. A wider range of well-characterized *Lactobacillus*-containing probiotics may improve bacterial vaginosis treatment outcomes, which would hopefully lower the burden of adverse pregnancy outcomes and sexually transmitted infections.

## Introduction

Maintenance of vaginal health is important in protecting women from adverse urogenital and reproductive health outcomes [1]. Optimally, the lower female genital tract (FGT) has a pH <4.5 [2,3] and a *Lactobacillus*-dominated microbiota [4,5]. However, the lower FGT microbiota frequently shifts to a non-optimal state that is characterised by a depletion of *Lactobacillus* spp. and high relative abundance of a diverse array of anaerobic bacteria, coinciding with elevation in vaginal pH ≥4.5—referred to as bacterial vaginosis (BV) [1]. BV can lead to severe reproductive complications [6–8], such as an increase in the risk for acquiring and transmitting sexually-transmitted infections (STIs) including human immunodeficiency virus (HIV) [9–11]. The current clinical standard of care (SOC) for BV is either oral or vaginal metronidazole or clindamycin [12]. However, antibiotic treatment of BV only results in a short-term cure as the recurrence rates are high, with ~50% of women recurring within six months [13,14]. As a result of this, several clinical studies evaluated *Lactobacillus*-containing probiotics as an adjunct to BV treatment and have shown largely beneficial although heterogeneous outcomes [14–17]. It is therefore crucial to develop more effective probiotic treatment strategies for BV. Few probiotics are explicitly marketed for vaginal health internationally and in South Africa [18], and only few of these contain *Lactobacillus* spp. commonly found in FGTs of women with optimal microbiota. Bacterial strains should fulfil specific biological criteria if their intended purpose is to be developed into a probiotic to improve FGT health–collectively referred to as the preferred product profile (PPP). In this study, we evaluated a range of PPP characteristics that should be considered in the development of vaginal probiotics. These included: (1) originating from the FGT, as vaginal *Lactobacillus* spp. are highly adapted for this specialized niche [19]; (2) ability to adhere well to FGT cells, as adherent isolates are more likely to remain locally [20–22]; (3) inhibiting the growth of BV-associated species, including *G*. *vaginalis* and *P*. *bivia*, [23–25] (4) tolerating low pH associated with vaginal health [26–28]; (5) lowering *in vitro* culture pH conditions, thereby inhibiting viral and bacterial pathogens [2,13,29,30]; (6) producing L- and D-lactate, which are involved in viral and bacterial pathogen inhibition [30–32]; and (7) tolerating antibiotics used to treat BV if administered in conjunction SOC [33–35]. All these PPP characteristics call for the use of bacterial strains adapted to the particular conditions of the FGT, which will mostly be met in *Lactobacillus* spp. originating from the FGT of generally healthy women. Thus, this study aimed to isolate and evaluate key characteristics of vaginal *Lactobacillus* strains from South African women to select the top performing strains for the development of a probiotic for vaginal health. Furthermore, the characteristics of vaginal strains were compared to those of commercially available probiotic strains currently used in probiotic formulations for vaginal health in South Africa and internationally.

## Results

A total of 57 *Lactobacillus* strains were isolated from the FGT of 26 young (median age 18 years; IQR 17–19 years) South African women, including 10 *L*. *crispatus*, 9 *L*. *gasseri*, 18 *L*. *jensenii*, 8 *L*. *vaginalis*, and 12 *L*. *mucosae* strains (Table 1). *Lactobacillus* isolates of the same species, derived from the same woman, were only included if they showed different *in vitro* characteristics to avoid selection of identical strains. The characteristics of these vaginal *Lactobacillus* strains were compared to four *Lactobacillus* ATCC reference strains (*L*. *crispatus* 33197, *L*. *gasseri* 9857, *L*. *jensenii* 25258, and *L*. *vaginalis* 49540) and 10 *Lactobacillus* strains isolated from commercially available probiotics, including *L*. *reuteri* (n = 1), *L*. *rhamnosus* (n = 6) and *L*. *acidophilus* (n = 3) (Table 1).

**Table 1:** Details of vaginal, probiotic and ATCC reference *Lactobacillus* strains.

Vaginal species	n	ID	Age^@^	Cluster^&^	BV*	STI^#^	Isolate ID
*L*. *crispatus*	10	70 80 94 95 96 100 73	19 18 18 17 17 18 16	- C2 None - - C2 -	- - - - - - +	- - - - - - -	70.1PA, 70.6PA 80.3a 94.77PA 95.34PA 96.9PA, 96.9PB, 96.27PA 100.16a 73.55a
*L*. *gasseri*	9	94 100 107 114 117	18 18 19 17 17	- C2 C3 C2 -	- - - Int. +	- - + + -	94.98PB 107.10PB, 107.7PA 100.5PA, 100.46PA 114.1PA, 114.30PA, 114.12PB 117.73PA
*L*. *jensenii*	18	88 94 95 96 72 93 84 73 89 92	20 18 17 17 16 17 18 16 18 18	- - - - C3 None C3 - - -	- - - - - - Int + + +	- - - - + + - - - -	88.10PA, 88.33PA 94.70PA 95.1PA, 95.22PA, 95.31PA, 95.37PA 96.8PA, 96.45PA, 96.45PB 72.14PA, 72.22PA 93.18PA 73.2PA 84.35PA 89.50PA 92.1PA, 92.27PA
*L*. *vaginalis*	8	80 88 91 100 73 81 79	18 20 18 18 16 18 18	C2 - C1 C2 - - C1	- - - - + + +	- - - - - - +	80.3b, 80.23b 88.5b 91.8a 100.13PA 73.27PA 81.17A 79.24PA
*L*. *mucosae*	12	80 99 85 102 87 98 86 90	18 20 16 17 19 21 22 18	C2 C1 - C3 C1 C3 - C1	- - Int Int + + + +	- - - - - - + +	80.23a 99.1PA 85.1PA, 85.30PA 102.33PA 87.5PA, 87.21PA 98.46PA, 98.52PA 86.4PA, 86.30PA 90.13PA
**ATCC strains** *L*. *crispatus* *L*. *gasseri* *L*. *jensenii* *L*. *vaginalis*	4						33197 9857 25258 49540
**Probiotic strains** *L*. *reuteri* *L*. *rhamnosus* *L*. *rhamnosus* *L*. *rhamnosus* *L*. *rhamnosus* *L*. *rhamnosus* *L*. *rhamnosus* *L*. *acidophilus* *L*. *acidophilus* *L*. *acidophilus*	10 Oral capsule Oral capsule Vaginal tablet Vaginal pessary Vaginal pessary Oral capsule Vaginal spray Oral capsule Vaginal spray Vaginal tablet						RFZ1006 (Reuterina) RFZ1006 (Reuterina) 0154 (Provacare) 0200 (Gynophilus) 7447212 (Muvagyn) C21134 (Vagiforte) S21134 (Vagiforte) C21134 (Vagiforte) S21134 (Vagiforte) T20868 (Vagiforte)

^@^Age in years

^&^C1- diverse microbiota, C2-*L*. *crispatus*-dominant, C3-*L*. *iners*-dominant; None-no cluster, -data n/a*by Nugent Scoring, with scores 0–3 being negative, 4–6 intermediate and 7–10 positive.

^#^including *C*. *trachomatis*, *N*. *gonorrhoeae*, *T*. *vaginalis*, *M*. *genitalium*, HSV-2, *H*. *ducreyi*, *T*. *pallidum* and *L*. *venerum*, ID-participant identification, CST-community-state-type

The majority of the South African women included in this study were BV and STI negative (Table 1), however, isolates from some women who were BV (n = 10) and/or STI (n = 6)-positive were included as well, to evaluate whether STI or BV status affects probiotic characteristics of vaginal *Lactobacillus* isolates. The vaginal microbiota of the participants belonged primarily to community cluster 1 (C1, diverse) and 3 (C3, *L*. *iners*-dominant), while only few belonged to cluster 2 (C2, *L*. *crispatus*-dominant) [36].

### Growth kinetics of vaginal and probiotic *Lactobacillus* isolates at differing pHs

**Fig 1:**
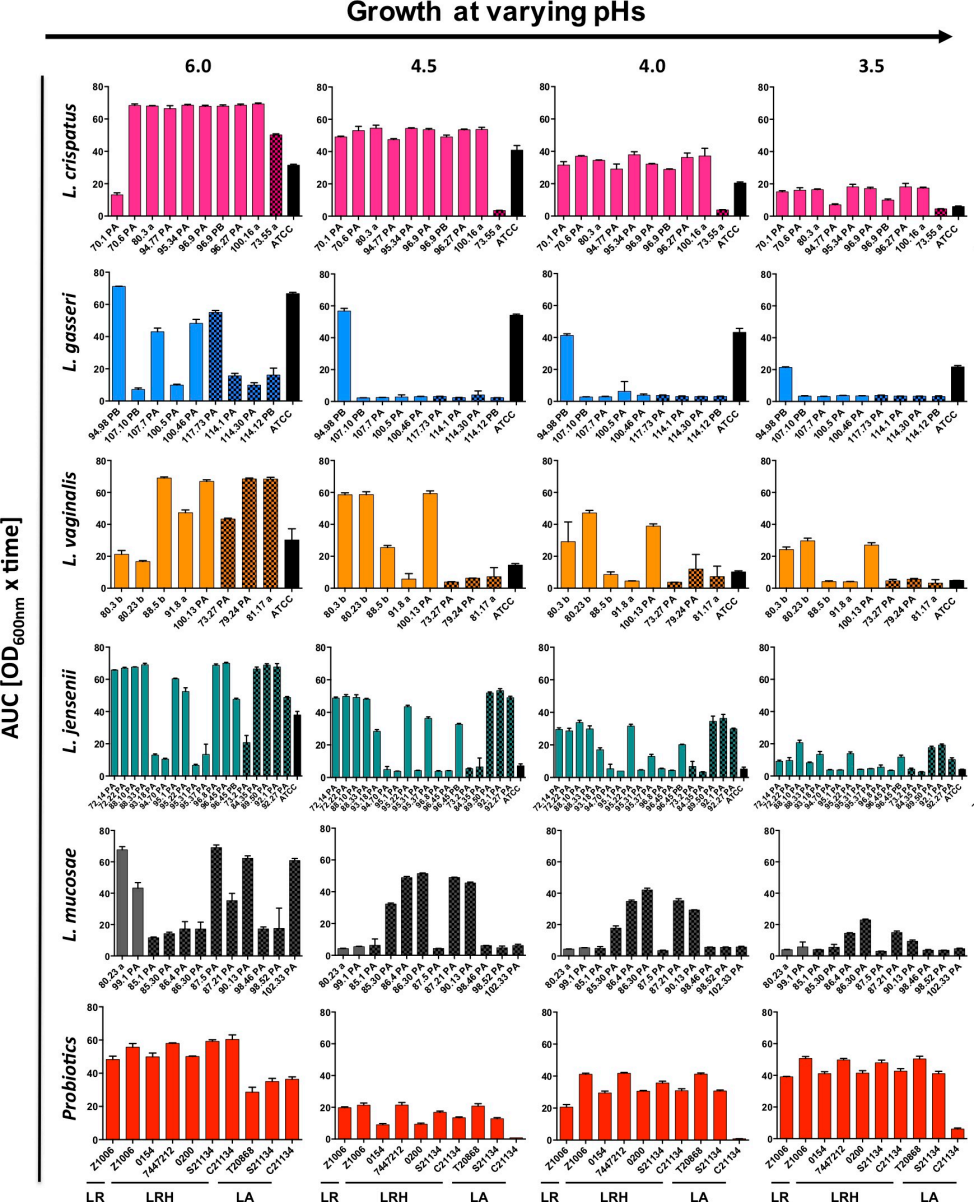
Growth of vaginal and probiotic *Lactobacillus* isolates at varying culture pHs. The growth of vaginal *L*. *crispatus* (pink), *L*. *gasseri* (blue), *L*. *vaginalis* (orange), *L*. *jensenii* (green) and *L*. *mucosae* (grey,) and commercially available probiotic strains (red, LR–*L*. *reuteri*, LRH = *L*. *rhamnosus*, LA = *L*. *acidophilus*) at varying pHs was measured over 48 hours. The AUC was calculated, and each bar represents the mean and standard deviation (SD) for each isolate. Isolates from BV/STI-negative women are shown by plain bars, those from BV and/or STI-positive women are patterned and ATCC strains are shown by black bars.

The *in vitro* growth kinetics of *Lactobacillus* strains under anaerobic conditions at pH 6.0 were evaluated, a characteristic that is important for commercial scale-up. The primary vaginal *Lactobacillus* isolates grew variably, between and within species (Fig 1). *L*. *crispatus* and *L*. *jensenii* strains grew the best, as quantified by calculation of the area under the curve (AUC), followed by *L*. *vaginalis*, *L*. *mucosae* and lastly *L*. *gasseri* strains.

The *in vitro* ability of isolates to grow at lower pHs was considered as important, since these conditions are optimal for the maintance of a healthy vagina [37,38]. All *L*. *crispatus* strains showed reduced growth at pHs 4.5, 4.0, and 3.5 compared to pH 6.0, although they appeared to tolerate low pHs better than other *Lactobacillus* spp. (Fig 1). In contrast, *L*. *jensenii*, *L*. *vaginalis*, *L*. *gasseri*, and *L*. *mucosae* strains appeared to be less acid tolerant, with the number of strains able to grow decreasing with lower pHs. Notably, there were no differences in growth at any pH between vaginal *Lactobacillus* strains isolated from women without BV or STIs versus those originating from women with BV and/or STIs (Fig 1). The majority of vaginal *L*. *crispatus*, *L*. *vaginalis* and *L*. *jensenii* strains grew better than their respective ATCC strain at all pHs tested. In comparison, the ten probiotic strains (including *L*. *reuteri*, *L*. *rhamonosus* and *L*. *acidophilus*) showed less growth than *L*. *crispatus* strains at pH 6.0, and the majority of probiotic strains showed some tolerance to pHs 3.5–4.5 (Fig 1).

### Ability to lower culture pH

**Fig 2:**
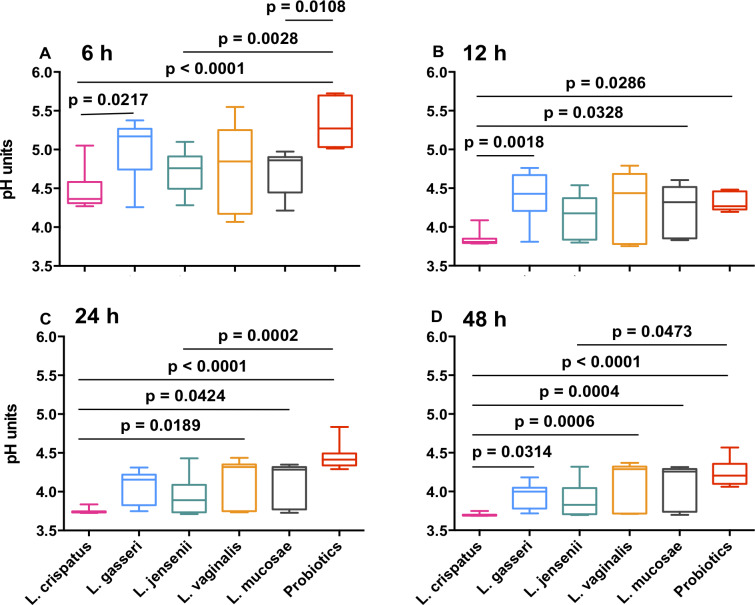
Ability of vaginal and probiotic *Lactobacillus* spp. to lower culture pH. MRS culture pH was measured over 48 hours for vaginal *L*. *crispatus* (pink), *L*. *gasseri* (blue), *L*. *jensenii* (green), *L*. *vaginalis* (orange), *L*. *mucosae* (grey) and the probiotic isolates (red; including *L*. *reuteri*, *L*. *rhamnosus* and *L*. *acidophilus* strains). Non-parametric multiple comparisons were done using the Kruskal Wallis test. P<0.05, after adjusting for multiple comparisons, are shown.

All vaginal *Lactobacillus* strains tended to have similar kinetics in their ability to lower pH under anaerobic conditions, with the lowest pH reached being pH 3.7, while none of the probiotic isolates lowered the pH <4.2 (S1 Fig). Overall, *L*. *crispatus* lowered the pH significantly more than *L*. *gasseri*, *L*. *mucosae*, *L*. *vaginalis* and any of the probiotic strains at 48 hours, after adjusting for multiple comparisons (Fig 2). Growth of individual isolates at pH 6.0 did not significantly predict their ability to lower culture pH at 48 hours (Spearman rho = -0.17, p = 0.165), indicating that differences in growth between strains did not account for differences in their ability to lower pH.

### L- and D-lactate production

**Fig 3:**
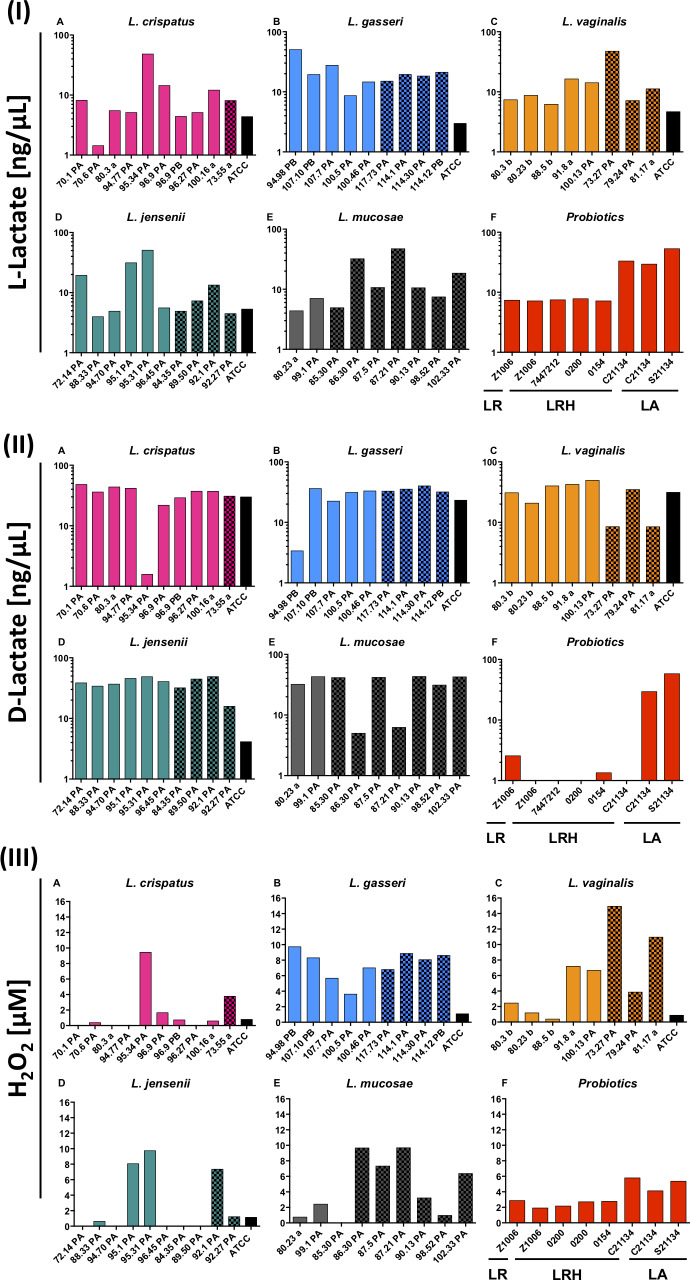
Ability of vaginal and probiotic *Lactobacillus* strains to produce (I) L-lactate, (II) D- lactate, and (III) H_2_O_2_ under anaerobic conditions. Concentrations of H_2_O_2_, L- and D-lactate produced by *L*. *crispatus* (A), *L*. *gasseri* (B), *L*. *vaginalis* (C), *L*. *jensenii* (D), *L*. *mucosae* (E) and the commercial probiotic (F; including *L*. *reuteri* [LR], *L*. *rhamnosus* [LRH] and *L*. *acidophilus* [LA]) strains after 24 hours anaerobic incubation were measured. Isolates from BV/STI-negative women are shown by plain bars, those from BV and/or STI-positive women are patterned and ATCC strains are shown by black bars.

L- and D-lactate are considered important metabolic by-products of *Lactobacillus* strain metabolism that have anti-viral and anti-bacterial activity, respectively [30–32]. Therefore, the ability of vaginal *Lactobacillus* strains (46/57, due to assay limitations) to produce L- and D-lactate were evaluated under anaerobic conditions (Fig 3A and 3B). Vaginal *Lactobacillus* spp. tended to produce lower amounts of L- than D-lactate (median 10.6 ng/μL [IQR 5.6–19.4] vs. 35.8 ng/μL [30.4–41.9], p<0.0001), although all appeared to produce both isomers. Some vaginal strains produced high levels of L- but almost no D-lactate (such as *L*. *crispatus* 95.34PA), while others produced large amounts of D- but little L-lactate (such as *L*. *jensenii* 96.45PA). Again, STI and BV status of the donor did not influence L- and D-lactate levels measured (Fig 3A and 3B). Further, the majority of vaginal *Lactobacillus* isolates produced more of both isomers than their respective ATCC reference strains. With the exception of two probiotic *L*. *acidophilus* isolates (which produced similar levels of both isomers), all probiotic strains produced higher levels of L- than D-lactate, although concentrations of both were significantly lower than those of the vaginal strains (p<0.0001).

The ability of strains to produce L- and D-lactate did not correlate (Spearman rho = 0.00, p = 0.9592), neither did production of D-lactate (Spearman rho = 0.05, p = 0.7250) or L-lactate (Spearman rho = -0.23, p = 0.0838) with growth. There was also no correlation between ability of isolates to lower culture pH and their ability to produce L- or D-lactate (Spearman rho = 0.15, p = 0.2604 for L-lactate; rho = -0.02, p = 0.8960 for D-lactate), indicating that lactate was not the only cause of lowering culture pH.

### H_2_O_2_ production

Earlier studies suggested that H_2_O_2_ was the most influential *Lactobacillus* spp.-produced metabolite to inhibit pathogens in the lower FGT, although these studies were conducted aerobically [39,40]. To mimic *in vivo* conditions in the lower FGT, all experiments in this study were performed under anaerobic conditions. All of the vaginal *L*. *gasseri* and *L*. *vaginalis* strains produced small quantities of H_2_O_2_ (ranging from 0.5–15 μM), while some of the *L*. *jensenii*, *L*. *crispatus*, and *L*. *mucosae* strains did not produce any measurable H_2_O_2_ at all (Fig 3C). Of all previously evaluated *in vitro* characteristics, H_2_O_2_ only correlated with L-lactate levels positively (Spearman rho = 0.61, p<0.0001), but not with D-lactate (Spearman rho = -0.20, p = 0.1504).

### Ability to inhibit *P*. *bivia* and *G*. *vaginalis* strains

**Fig 4:**
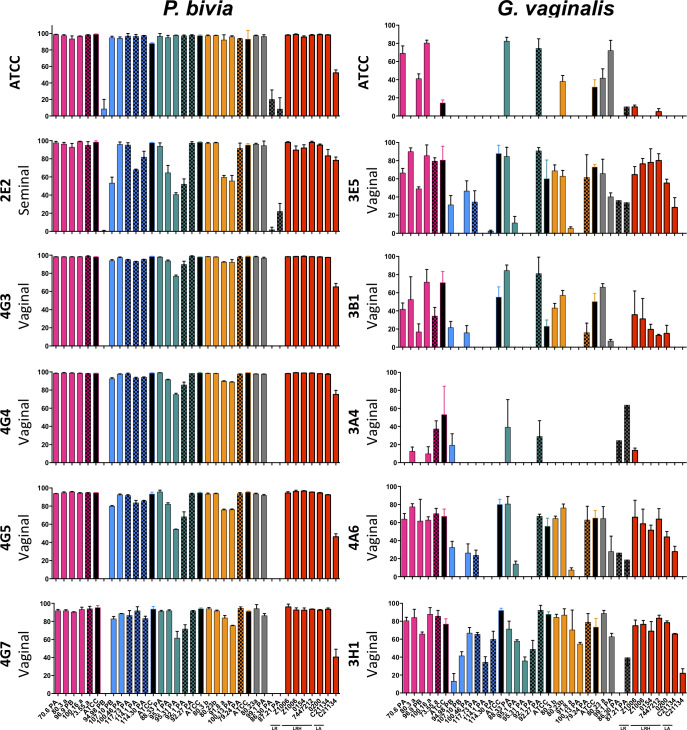
Ability of *Lactobacillus*-conditioned media to inhibit the growth of *P*. *bivia* and *G*. *vaginalis*. Conditioned media from *L*. *crispatus* (pink), *L*. *gasseri* (blue), *L*. *jensenii* (green), *L*. *vaginalis* (orange), *L*. *mucosae* (grey) and probiotic (red, LR–*L*. *reuteri*, LRH = *L*. *rhamnosus*, LA = *L*. *acidophilus*) cultures were co-incubated with reference ATCC and genital *P*. *bivia* and *G*. *vaginalis* strains for 48 hours and the AUC was calculated for each strain. The percentage of inhibition was determined by comparing the growth of *P*. *bivia* and *G*. *vaginalis* strains in the presence of non-conditioned control media to *Lactobacillus*-conditioned media. Isolates from BV/STI-negative women are shown by plain bars, those from BV and/or STI-positive women are patterned and ATCC strains are shown by black bars.

The ability of abiotic culture supernatants from a subset of vaginal *Lactobacillus* strains (25/57; selected based on performance in previous assays) to inhibit the growth of a panel of *P*. *bivia* and *G*. *vaginalis* strains was determined under anaerobic conditions (Fig 4). Most of the vaginal *Lactobacillus* isolates (besides *L*. *gasseri* 94.98PB, *L*. *mucosae* 86.30PA and 87.21PA) showed some degree of inhibition towards all clinical *P*. *bivia* strains, although the extent of inhibition differed by *Lactobacillus* and *P*. *bivia* strain (Fig 4). *L*. *crispatus* tended to inhibit *P*. *bivia* growth to a greater extent than other species, inhibiting all *P*. *bivia* strains > 90%. Vaginal *Lactobacillus* strains inhibited *P*. *bivia* similarly to their respective ATCC and the probiotic *Lactobacillus* strains (Fig 4).

In contrast, the inhibition of *G*. *vaginalis* strains was generally more variable and significantly lower (Fig 4). Only three vaginal *Lactobacillus* (*L*. *crispatus* 100.16A, *L*. *jensenii* 88.33PA and 92.27PA) and the *L*. *crispatus* ATCC strain inhibited the growth of the full panel of *G*. *vaginalis* strains. Inhibition appeared to be highly *G*. *vaginalis* strain-specific, as almost all *Lactobacillus* strains inhibited the vaginal *G*. *vaginalis* isolate 3H1 (Fig 4; bottom row), while only 9/25 inhibited the *G*. *vaginalis* ATCC strain (Fig 4; top row). Overall, *L*. *crispatus* strains tended to exhibit the strongest inhibitory activity against *G*. *vaginalis*, although this was not statistically significant. The ATCC reference *Lactobacillus* strains generally showed broader and better inhibitory activity against *G*. *vaginalis* than vaginal *Lactobacillus* isolates (Fig 4). Notably, the abilities of vaginal *Lactobacillus* strains to inhibit the pathogens tested were highly correlated (*G*. *vaginalis* and *P*. *bivia*: Spearman rho = 0.814, p<0.0001), indicating that individual *Lactobacillus* strains have a similar capacity to inhibit various BV-associated bacteria. Similarly to the previously assessed characteristics, BV/STI status of the donor did not influence inhibitory abilities.

### Antibiotic susceptibility

**Fig 5:**
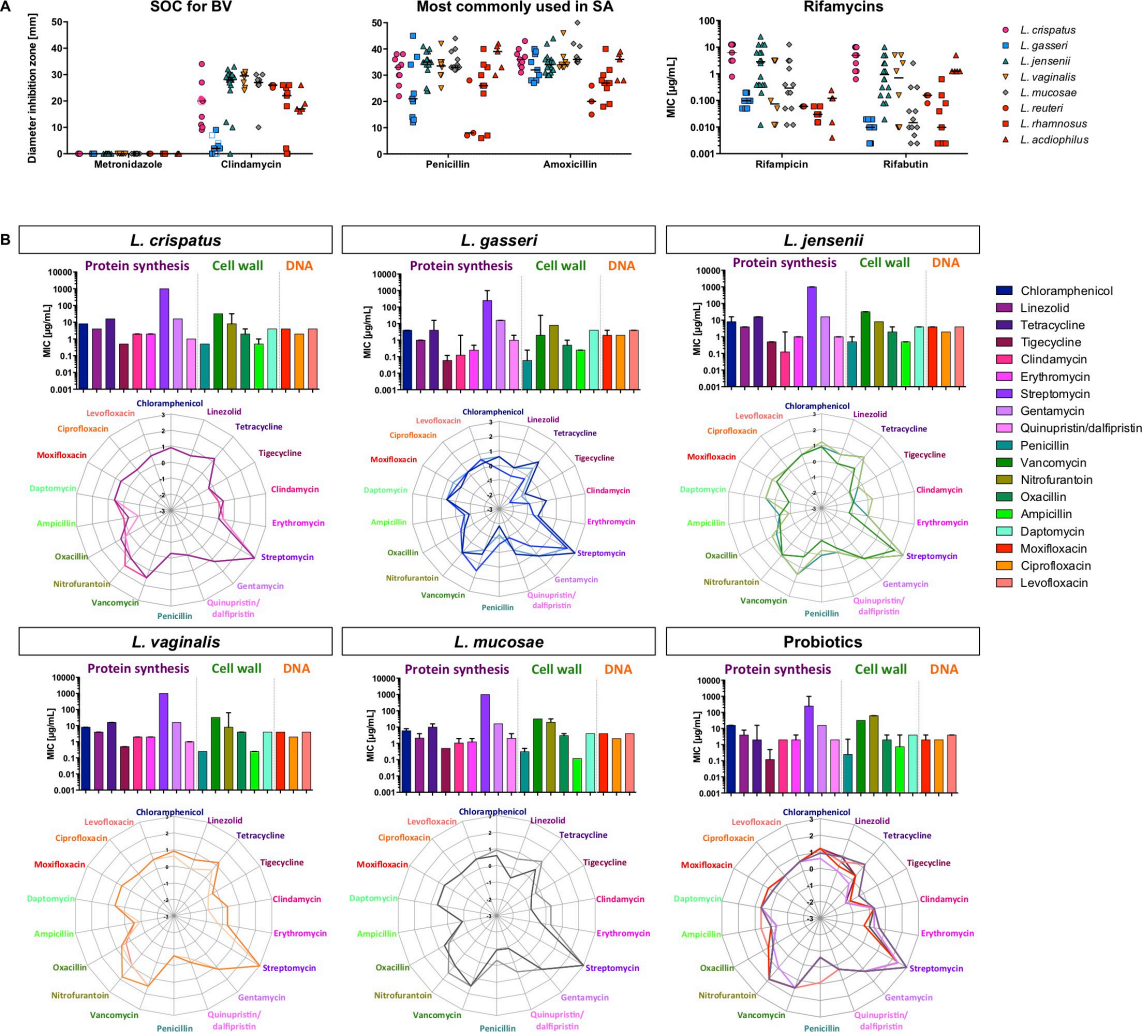
Antibiotic susceptibility of vaginal and probiotic *Lactobacillus* isolates. (A) The susceptibility of *L*. *crispatus* (pink), *L*. *gasseri* (blue), *L*. *jensenii* (green), *L*. *vaginalis* (orange), and *L*. *mucosae* (grey) and probiotic (red) isolates to metronidazole (5 μg), clindamycin (2 μg), penicillin (2 μg) and amoxicillin (10 μg) was determined using disc diffusion assays. The susceptibility to rifampicin and rifabutin was determined using broth dilution assays. (B) For a subset of 20 *Lactobacillus* isolates, the MIC to antibiotics interfering with the protein synthesis (purple), cell wall and membrane (green) and DNA synthesis (red) was determined using Sensititre plates. Bar graphs show the median (IQR) for each species, while each line in the radar plot represent the susceptibility (log-transformed MICs) of individual isolates.

Metronidazole and clindamycin are SOC for BV treatment and penicillin and amoxicillin are commonly used antibiotics. All vaginal *Lactobacillus* isolates (n = 57) were resistant to metronidazole, while the susceptibility to clindamycin varied within and between *Lactobacillus* spp. (Fig 5A). *L*. *vaginalis* and *L*. *jensenii* isolates were the most susceptible to clindamycin, while *L*. *gasseri* strains were the least. The inhibition zones of the probiotic strains were smaller than those of most clinical strains (with the exception of *L*. *gasseri*), indicating that these might be less susceptible to clindamycin than the majority of vaginal *Lactobacillus* strains.

All vaginal *Lactobacillus* isolates showed similar susceptibility to penicillin, with the exception of *L*. *gasseri* isolates, which tended to be less susceptible (Fig 5A). Probiotic *L*. *acidophilus* strains were more susceptible to penicillin than most vaginal strains, while *L*. *rhamnosus* isolates tended to be similarly susceptible, and *L*. *reuteri* less. The inhibition zones for amoxicillin were highly comparable to those measured for penicillin, as was the intra-species variability (Fig 5A). *L*. *crispatus* isolates were significantly more susceptible to amoxicillin than *L*. *rhamnosus* (adj. p = 0.0497), and *L*. *mucosae* than *L*. *reuteri* (adj. p = 0.0128) and *L*. *rhamnosus* (adj. p = 0.0033), indicating that *L*. *reuteri* and *L*. *rhamnosus* but not *L*. *acidophilus* strains may be less susceptible to amoxicillin than the vaginal isolates.

The rifamycins rifampicin and rifabutin are commonly used to treat tuberculosis (TB) and form part of the six-month antibiotic regimen administered to ~500,000 people in South Africa who develop TB annually [41], and have previously been explored as a treatment option for BV [42,43]. All *Lactobacillus* isolates were highly susceptible to rifampicin (<12.5 μg/ml) and rifabutin (<10.0 μg/mL) and susceptibilities of the probiotic isolates were similar to the vaginal strains (Fig 5A).

Broader antibiotic resistance profiling to inhibitors of protein, cell wall/membrane and DNA synthesis was conducted on a subset of 14 vaginal and six probiotic *Lactobacillus* isolates (Fig 5B). Vaginal *L*. *gasseri* strains appeared to be susceptible to a wider range of antibiotics than other *Lactobacillus* spp. *L*. *crispatus* strains showed exactly the same antibiotic susceptibility, while the intra-species variability was higher for other vaginal *Lactobacillus* spp. There were no clear differences between vaginal and probiotic *Lactobacillus* spp. in antibiotic susceptibility profiles. Based on the clinical MIC breakpoints defined by the EUCAST (2019), all vaginal isolates would be considered sensitive to ampicillin (MIC ≤4 μg/mL), and the majority to chloramphenicol (MIC ≤8μg/mL). For penicillin, (sensitivity MIC≤0.25 μg/mL, resistance MIC >0.5μg/mL), all clinical *L*. *gasseri*, *L*. *mucosae* and *L*. *vaginalis* strains were sensitive, all *L*. *crispatus* strains and *L*. *jensenii* 88.33PA strains had an intermediate sensitivity, and *L*. *jensenii* 92.27PA was resistant. *L*. *crispatus* 100.16A, *L*. *gasseri* 117.73PA and 100.46PA, *L*. *jensenii* 92.17PA and 95.31PA, *L*. *vaginalis* 79.24PA and *L*. *mucosae* 102.33PA were susceptible to clindamycin (MIC ≤1 μg/mL), while all other isolates had MICs >1μg/mL. Since the breakpoint for clindamycin has been defined as >4μg/mL, accoding to the EUCAST 2019 guidelines, it was not possible to conclude with the current data whether these isolates are resistant.

### Adhesion to ectocervical cells and inflammatory responses

**Fig 6:**
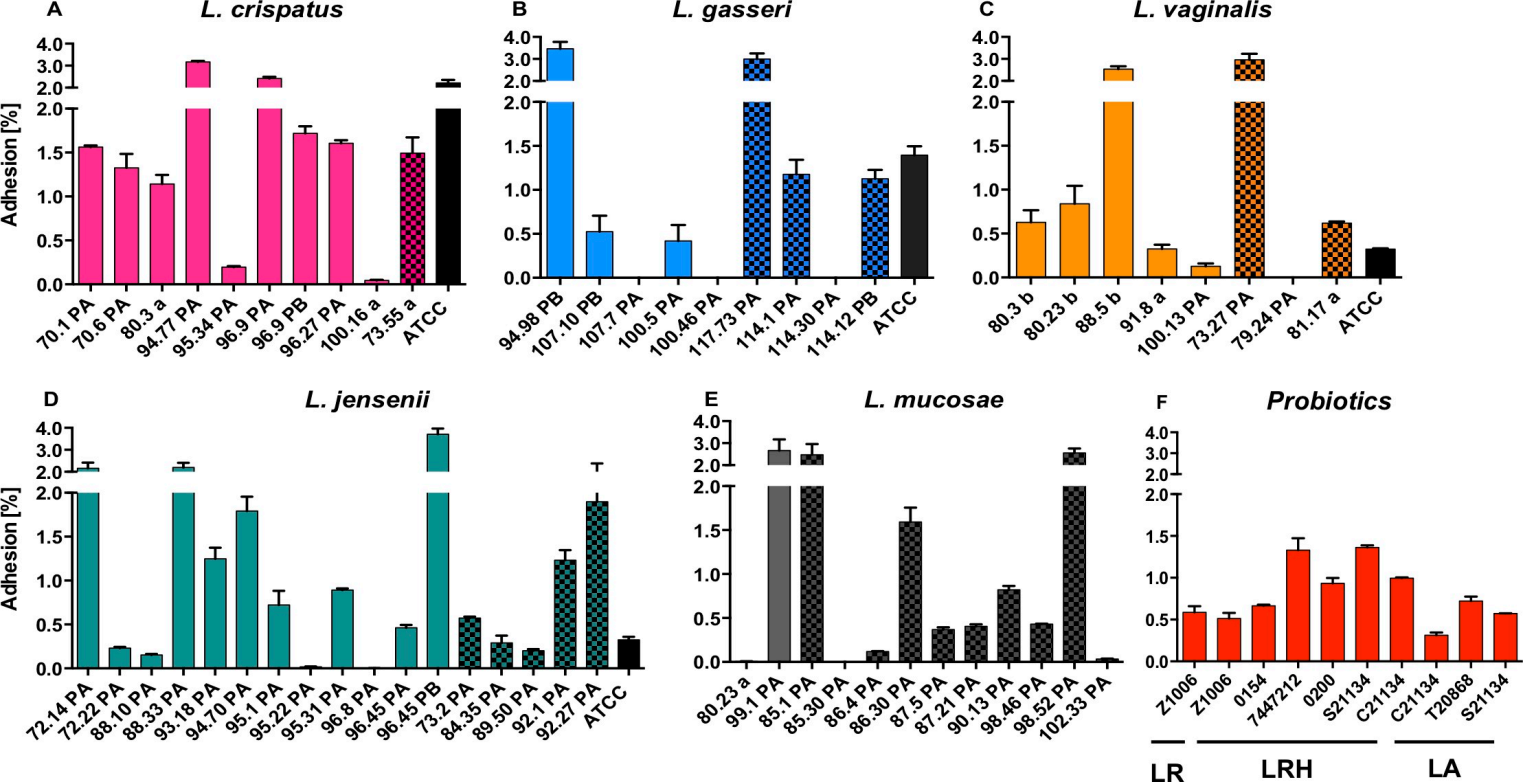
Adhesion of vaginal and probiotic *Lactobacillus* isolates to ectocervical Ca Ski cells. *Lactobacillus* isolates were co-cultured with Ca Ski cells for 3 hours and the percentage of adhered bacteria was calculated for *L*. *crispatus* (A), *L*. *gasseri* (B), *L*. *vaginalis* (C), *L*. *jensenii* (D), *L*. *mucosae* (E) and the commercially available probiotic isolates (F, LR = *L*. *reuteri*, LRH = *L*. *rhamnosus*, LA = *L*. *acidophilus*). Isolates from BV/STI-negative women are shown by plain bars, those from BV and/or STI-positive women are patterned and ATCC strains are shown by black bars.

The selected *Lactobacillus* strains should strongly adhere to host cells [21,22] but not influence cell viability or cause inflammatory changes [44,45]. All vaginal *L*. *crispatus* (10/10), and the majority of *L*. *vaginalis* (7/8), *L*. *jensenii* (16/17) and *L*. *mucosae* (10/12) and two-thirds of the *L*. *gasseri* (6/9) strains adhered to Ca Ski cells to varying extents, independently of the BV/STI status of the donor (Fig 6). Overall, vaginal *L*. *crispatus* strains tended to adhere the strongest followed by *L*. *mucosae*, *L*. *jensenii*, *L*. *vaginalis*, and *L*. *gasseri*, and several vaginal isolates (2/10 *L*. *crispatus*, 2/9 *L*. *gasseri*, 6/8 *L*. *vaginalis*, and 11/18 *L*. *jensenii*) adhered better than their respective ATCC reference strain (Fig 6).

**Fig 7:**
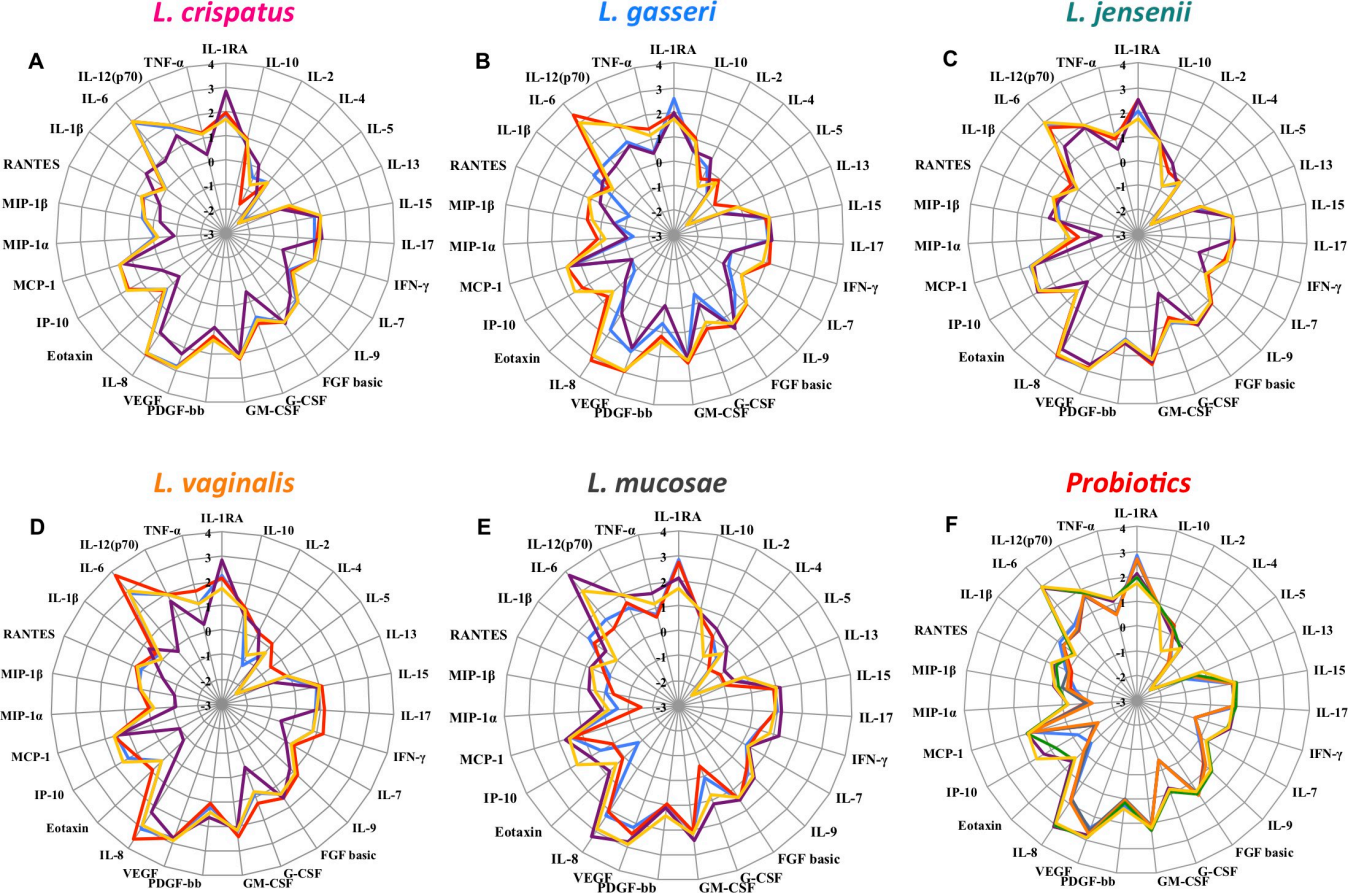
Vaginal Ca Ski cell cytokine responses following co-culture with vaginal and probiotic *Lactobacillus* isolates. The concentrations of 27 cytokines were measured in culture supernatants by Luminex. Abiotic cell culture supernatant was used as control (shown in yellow). The log-transformed concentrations of all measured cytokines for each three (shown in purple, blue and red) selected *L*. *crispatus* (A), *L*. *gasseri* (B), *L*. *jensenii* (C), *L*. *vaginalis* (D) and *L*. *mucosae* (E) and six probiotic (F) isolates are shown in radar plots. While all data points outside of the yellow radar (control) show an upregulation of cytokines, those inside the yellow radar indicate a downregulation of a certain cytokine.

For a subset of vaginal *Lactobacillus* isolates that included three strains per vaginal *Lactobacillus* spp. and six probiotic strains, the effect on cervical epithelial Ca Ski cell viability and cytokine expression was determined (Fig 7). None of the vaginal strains decreased the viability of Ca Ski cells below 80%, as measured by trypan blue straining. After grouping the cytokines into their biological categories, all bacterial isolates up-regulated regulatory cytokines (including IL-1RA and IL-10) 1.5-fold (*L*. *crispatus* 100.16 a) to 10-fold (*L*. *reuteri* Z1006) compared to the control. In contrast, only five *Lactobacillus* strains (*L*. *gasseri* 100.46 PA, *L*. *mucosae* 80.23 a, *L*. *vaginalis* 79.24 PA, *L*. *rhamnsus* P0154 and C21134) induced adaptive cytokines (including IL-2/4/5/13/15/17 and IFN- γ) ~ 2-fold more than the control, while the remaining 16 strains downregulated adaptive cytokines up to 1.6-fold. Only for *L*. *gasseri* 100.46 PA slightly higher levels of growth factors and haematopoietic cytokines (including IL-7/9, FGF-basic, G-CSF, GM-CSF, PDGF-BB, and VEGF) were measured compared to the control, and the remaining strains downregulated this group of cytokines up to 4-fold. For about half of the strains (10/21) up to 4.2-fold higher levels of chemokines (including IL-8, Eotaxin, IP-10, MCP-1, MIP-1α/β and RANTES) were measured than for the control, while for the other half up to 24-fold lower levels were found. Four strains (*L*. *gasseri* 100.46 PA, *L*. *mucosae* 80.23 a, *L*. *vaginalis* 79.24 PA, *L*. *rhamnosus* C21134) induced higher inflammatory responses (1.02-, 2.44-, 5.84-, 5.88-fold, respectively, including IL-1β/6/12(p70) and TNF-α) than the control, while the remaining 17 *Lactobacillus* strains downregulated inflammatory responses up to 47-fold. In summary, the majority of bacterial strains induced cytokine responses comparable to the control and did not induce inflammatory responses. However, for *L*. *gasseri* 100.46 PA, *L*. *mucosae* 80.23 and *L*. *vaginalis* 79.24 PA higher levels of cytokines were measured in all cytokine categories, indicating that the inflammatory potential of these *Lactobacillus* strains requires further investigation.

### Assessment of overall PPP performance

**Fig 8:**
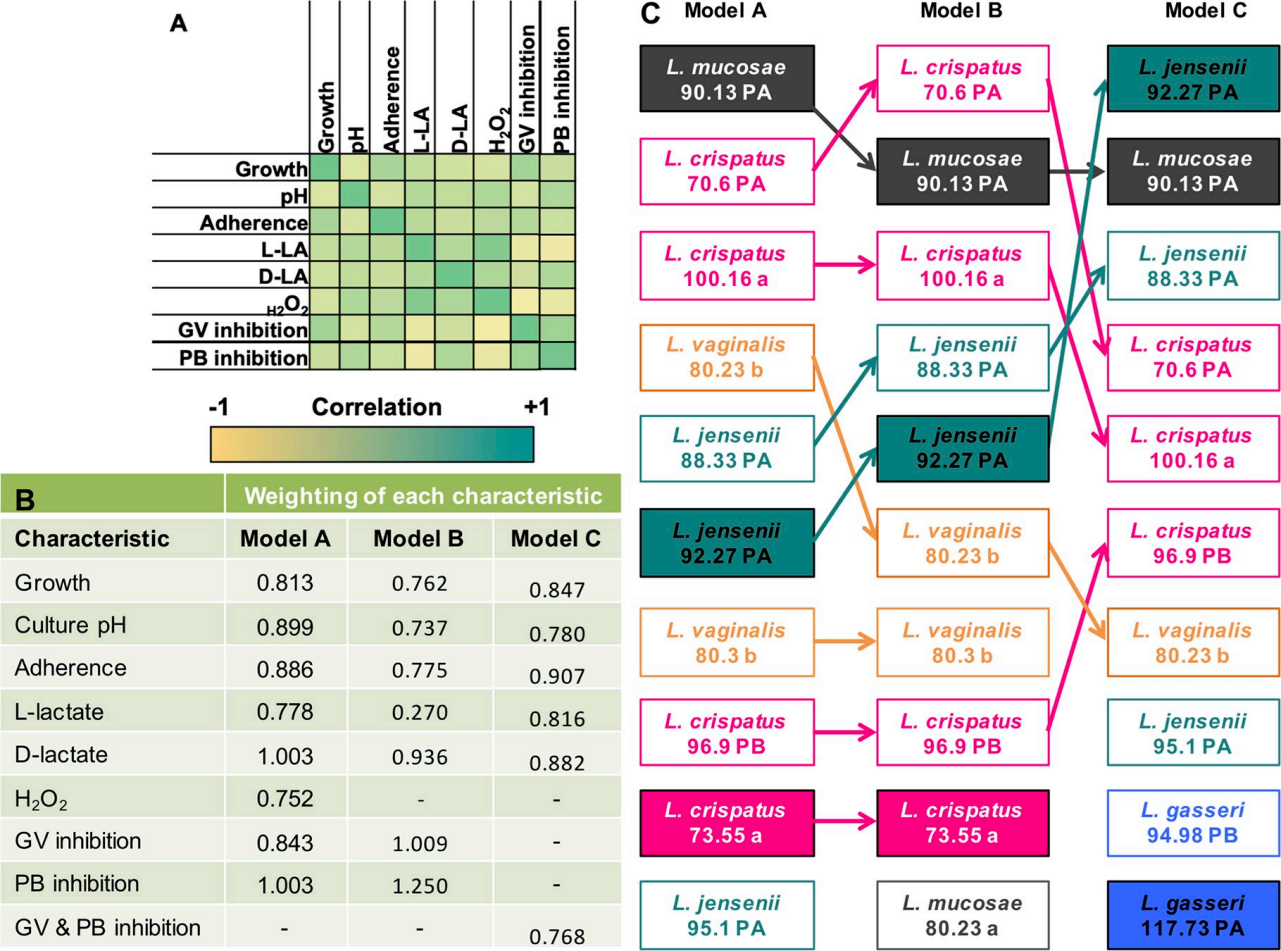
Selection of top-performing of *Lactobacillus* strains. Correlation matrix of measured characteristics (A); and weighting of each characteristic in four different ranking models (A-C) that were considered (B). Model A included all characteristics. Model B excluded H_2_O_2_ production (as this may not be biologically relevant under anaerobic conditions) and Model C excluded H_2_O_2_ production in addition to grouping *G*. *vaginalis* and *P*. *bivia* inhibition into one category (since pathogen inhibition correlated strongly). Dark green indicates a strong positive correlation, while dark yellow indicates a strong negative correlation. (C) Relative ranking of vaginal *Lactobacillus* isolates according to the three different models that were considered. Species are color-coded (*L*. *crispatus* = pink, *L*. *gasseri* = blue, *L*. *jensenii* = green, *L*. *vaginalis* = orange, and *L*. *mucosae* = grey). Filled boxes indicate which strains were obtained from BV and/or STI-positive women.

To identify the most promising vaginal *Lactobacillus* strains for the development of a probiotic for vaginal health, a weighted scoring system was devised based on PPP characteristics, including: their ability to grow well *in vitro*, lower pH, adhere to cervical epithelial cells, produce L- and D-lactate and H_2_O_2_, and inhibit growth of BV-associated species (*G*. *vaginalis* and *P*. *bivia*). Cytokine and antibiotic susceptibility profiles were not considered, as data were available only for a subset of vaginal strains. For this ranking, a total of 36/57 *Lactobacillus* strains (for which complete characteristic evaluations were available) were included. To allow objective comparisons, the performance of vaginal isolates was compared with their respective ATCC reference and the probiotic isolates. Because some of the characteristics included in the ranking correlated, a weighted scoring system was developed to account for co-linearity between characteristics (Fig 8A). Including all characteristics in the scoring matrix (**model A;** Fig 8B), *L*. *mucosae* 90.13PA, *L*. *crispatus* 70.6PA and 100.16A, *L*. *vaginalis* 80.23B, and *L*. *jensenii* 88.33PA and 92.27PA were the six highest scoring *Lactobacillus* strains, with similar points as the ATCC *L*. *crispatus* and ATCC *L*. *gasseri* strains, while all commercially available probiotics ranked lower. Further step-wise sensitivity analyses were performed to test the robustness of the selection. **Model B:** Because the concentrations of H_2_O_2_ produced by *Lactobacillus* were all below the level found to be microbicidal [30], a ranking model was considered in which H_2_O_2_ was excluded, which did not change the ranking of the top six strains (Fig 8C). **Model C:** In addition to excluding H_2_O_2_ production, the inhibitory effect on *G*. *vaginalis* and *P*. *bivia* were grouped to avoid skewing towards strains that performed generally well in the inhibition assays (Fig 8C), which resulted in two *L*. *gasseri* strains being included in the top-10 strains.

The sensitivity analysis suggested that the scoring system was quite robust and showed that isolates from both STI/BV-negative and women with STIs or BV were among the top-performing strains, indicating that even *Lactobacillus* strains that originate from women with BV or STIs can have promising probiotic characteristics. Although all commercially available probiotic strains were included in the scoring system, none scored in the top ten in any of the models considered.

### WGS analysis of selected *Lactobacillus* strains

Prior to selecting vaginal *Lactobacillus* strains for the development of a probiotic, we selected well-performing strains from a range of *Lactobacillus* spp. for whole genome sequencing and genome analysis to assess the presence of antimicrobial resistance determinants, mobile elements and prophages, including *L*. *mucosae* 90.13PA (ranked consistently in the top 2, from BV/STI-positive women), *L*. *crispatus* 70.6PA (highest ranking *L*. *cripsatus* strain from BV/STI-negative women) and *L*. *crispatus* 73.55a (highest ranking *L*. *cripsatus* strain from BV-positive women), *L*. *gasseri* 94.98PB (highest ranking *L*. *gasseri* strain, from BV/STI-negative women), and *L*. *jensenii* 95.1PA (from BV/STI-negative women).

The whole genomes of five selected strains were sequenced to a median depth of 117X (range 102-142X). Full assembly statistics are shown in S1 Table. Screening for the presence of well characterised antimicrobial resistance determinants revealed no matches to the Resfinder, CARD and AMRFinder databases for any of the assemblies. However, analysis of the RAST annotated assemblies revealed that all five strains harboured open reading frames (ORFs) with homology to the *ldb0660* and *copY*-like genes in *Lactobacillus delbrueckii* subsp. *bulgaricus* that respectively encode a copper-translocating P-type ATPase and a negative transcriptional regulator, both of which play a role in copper homeostasis. In addition, three of the five strains (*L*. *crispatus* 70.6 PA, *L*. *crispatus* 73.55 a and *L*. *mucosae* 90.13 PA) harboured genes encoding putative TetM-type ribosomal protection proteins. Two strains (*L*. *gasseri* 94.98 PB and *L*. *jensenii* 95.1 PA) possessed potential beta-lactamase class A proteins, while *L*. *crispatus* 73.55 a harboured an ORF encoding a putative streptothricin acetyltransferase. The latter was flanked by genes encoding predicted mobile element-associated transposases. Finally, two classes of potential multidrug transporters were identified. All strains except *L*. *gasseri* 94.98 PB harboured genes encoding putative multidrug and toxin extrusion (MATE) family efflux pumps with homology to the MepA protein in *Staphylococcus aureus* and a further three strains (*L*. *crispatus* 70.6 PA, *L*. *crispatus* 73.55 a and *L*. *jensenii* 95.1 PA) harboured genes encoding putative major facilitator superfamily (MFS) class efflux pumps with homology to the Lde efflux pump in *Listeria monocytogenes*.

Putative intact prophages were present in the genomes of two of the five strains. *L*. *crispatus* 70.6 PA harboured a single predicted 43.9Kbp prophage, while *L*. *mucosae* 90.13 PA harboured three predicted prophages of 16.9Kbp, 37.5Kbp and 40.1Kbp, respectively. Complete CRISPR-Cas loci (i.e. one or more signature *cas* genes located alongside a CRISPR array) were identified in the genomes of three of the strains. *L*. *crispatus* 73.55 a harboured a type I-E locus with spacers showing matches to a lactococcal phage Tuc2009. *L*. *mucosae* 90.13 PA harboured two distinct loci encoding a type I-C and type III-A system, respectively. Spacers present in the type I-C locus showed homology to phage-like regions on the *L*. *mucosae* LM1 plasmid and the *Streptococcus thermophilus* phage Sfi19, while those in the type III-A locus showed homology to several plasmids (*L*. *mucosae* LM1, *C*. *botulinum* pCBH, and an unnamed plasmid in *Lactobacillus fermentum* DR9) as well as the *Streptococcus* phage Javan623. *L*. *jensenii* 95.1 PA harboured a single type II-A locus with spacers showing homology to several phages (an enterococcal phage, EFRM31 and the *Lactobacillus* phages Lf1 and Lj928).

## Discussion

A limited variety of probiotics for vaginal health are currently available internationally and in South Africa, and few of these contain FGT commensals [18]. Thus, there is an urgent need for the development of additional well-designed probiotics for vaginal health. In this study, we evaluated key probiotic characteristics of a large panel of vaginal *Lactobacillus* strains, including *L*. *crispatus*, *L*. *gasseri*, *L*. *jensenii*, *L*. *vaginalis* and *L*. *mucosae*, from South African women. *L*. *iners* was not included, since its role in FGT health and disease remains unclear [46]. Overall, the majority of vaginal *Lactobacillus* strains performed better according to the PPP criteria considered than the included currently available commercial probiotic strains.

All *Lactobacillus* strains grew optimally at pH 6.0, similar to what has been described previously [28], despite existing at pHs <4.5 in the FGT [36,47] and actively lowering culture pH. All vaginal *L*. *crispatus* strains lowered the pH *in vitro* to 3.7, while only some strains of other vaginal *Lactobacillus* spp. achieved pHs <4.0. *In vivo*, women with an *L*. *crispatus*-dominated vaginal microbiota have been shown to have a lower FGT pH than women with a *L*. *gasseri-* or *L*. *jensenii*-dominated microbiota [4], suggesting good alignment between these *in vitro* characteristics and *in vivo* phenotypes.

The ability to produce lactate varied between *Lactobacillus* spp. and strains, although vaginal *L*. *jensenii* strains produced the lowest L-lactate (close to detection limit) concentrations, which supports previous studies showing that *L*. *jensenii* strains commonly only produce D-lactic acid [48,49]. Hutt et al. (2016) also found that *L*. *gasseri* produced significantly more lactate than *L*. *jensenii* and *L*. *crispatus*, which agrees with our findings. No correlation between lactate production and culture pH was found, but it has been suggested that other acids, such as acetic acid, contribute to lowering pH [50]. Under anaerobic conditions, vaginal *Lactobacillus* strains produced <20μM H_2_O_2_. Considering that the microbicidal activity of H_2_O_2_ has only been demonstrated at concentrations ≥1000 mM [30], H_2_O_2_ production by vaginal *Lactobacillus* spp. seems to be biologically irrelevant [51].

Vaginal and probiotic *Lactobacillus* strains inhibited *G*. *vaginalis* growth in a highly strain-specific manner. This indicates that *G*. *vaginalis* genotype may influence susceptibility to *Lactobacillus*-mediated inhibition. Previous studies have shown that metronidazole-resistant *G*. *vaginalis* strains were less susceptible to inhibition by *Lactobacillus* spp. [23,52], and biofilm-producing *G*. *vaginalis* strains were more tolerant to lactic acid than non-biofilm producing strains [53]. As such, *G*. *vaginalis* strain 3H1, which was inhibited broadly and strongly, belongs to genotype B, is not resistant to metronidazole and a low biofilm producer, which might explain the high susceptibility to *Lactobacillus*-mediated inhibition. In contrast, *G*. *vaginalis* 3B1 that showed low levels of inhibition belongs to genotype C and is highly resistant to metronidazole, which might explain why it was less susceptible to inhibition by *Lactobacillus* spp.

Antibiotic susceptibility has an impact on whether the probiotic could be administered concurrently with SOC [54]. All vaginal *Lactobacillus* strains were resistant to metronidazole, as expected, and indeed, concentrations between 128 and 256 μg/ml of metronidazole have been shown to stimulate the growth of vaginal *Lactobacillus* spp. *in vitro* [55]. Few *Lactobacillus* strains were resistant to clindamycin, as described by others [56–58], indicating that these strains could not be administered concurrently with SOC clindamycin treatment. Of note, all *Lactobacillus* strains were highly sensitive to rifamycins. Considering that these are administered to ~500,000 people annually in South Africa for six months to treat TB, effects of long-term administration of rifamycins on the FGT microbiota should be evaluated.

It is also important that probiotic candidates do not cause pathology to the host, such as an increase in genital inflammation [44], especially in a setting with extremely high STI and HIV rates like in South Africa. Promisingly, we found that all vaginal *Lactobacillus* strains up-regulated the regulatory cytokine IL-1RA, which inhibits binding of pro-inflammatory cytokines like IL-1α and IL-1β to their cognate receptors [59]. Some of the *Lactobacillus* strains even increased IL-8 production, which is important to consider, as IL-8 has been found to decrease HIV-1 transcription in both lymphocytes and ectocervical tissue explants [60]. Only four strains induced the inflammatory cytokines TNF-α, IL-12(p70), IL-6 and IL-1β, while the remaining *Lactobacillus* strains downregulated these, which is again reassuring as genital inflammation has been linked to HIV and STI risk [61]. Other cytokines that have been related to the risk of HIV acquisition in South African women include MIP-1α, MIP-1β and IP-10 [62], and it is encouraging that all besides three *Lactobacillus* strains downregulated these. Together, these findings indicate that administration of most vaginal *Lactobacillus* strains evaluated here is likely to be considered safe in a population with high STI and HIV prevalence but this needs confirmation in preclinical animal models and small interventional trial in healthy humans.

Using a weighted PPP scoring system, the identified top-performing strains included only vaginal *Lactobacillus* strains, obtained both from BV/STI negative South African women as well as some from donors who had BV and/or STIs, while none of the commercially available strains ranked as well, suggesting that there is potential to improve currently available probiotic formulations for vaginal health. Surprisingly, we did not find that strains from BV and STI-negative women consistently performed better than those from BV/STI-positive women, indicating that the probiotic potential of vaginal *Lactobacillus* strains is not necessarily dependent on the vaginal health of the donor.

The WGS of five of the best-performing vaginal *Lactobacillus* strains further confirmed the likelihood of their safety, due to antimicrobial resistance elements being largely absent. *L*. *crispatus* 73.55a harboured a streptothricin acetyltransferase sequence flanked by mobile elements, which is concerning as this could be one potential mechanism leading to the transfer of streptothricin resistance [63]. Prophage sequences were also identified, in agreement with other studies that observed functional and non-functional integrated bacteriophages in vaginal *Lactobacillus* spp. isolates [64–66]. While the relevance of potentially functional prophages for vaginal health is still unclear, it may be necessary to ensure that these prophage sequences are non-functional prior to inclusion of the strains into a commercial probiotic.

Several limitations of our study need to be acknowledged. No information on the strains that were included in the commercial probiotics were made available by all manufacturers. Not all characteristics were determined for all 57 strains, but we selected strains for further characterisation based on evidence of promising PPP characteristics. We also did not evaluate the mechanisms of pathogen inhibition.

In summary, this study evaluated probiotic characteristics of numerous vaginal *Lactobacillus* strains from South African women. The probiotic potential of these isolates was found to be strain-specific, and vaginal *Lactobacillus* strains largely performed better than probiotic strains currently used in probiotics for vaginal health, available internationally and in South Africa. Some of the best-performing, sequenced vaginal *Lactobacillus* isolates are now being considered for the development of a probiotic for vaginal health. This probiotic will be vaginally administered because vaginal administration affects vaginal health more quickly than does oral administration and enhances the viability of the administered microorganism [67]. It remains to be decided whether this probiotic should be region-specific, and only available to Southern African women due to the origin of its *Lactobacillus* strains, or worldwide. Once its effect on BV cure and recurrence has been tested in South Africa and found to be effective, the probiotic should also be tested in non-African women to determine whether these *Lactobacillus* strains show a similarly beneficial effect in other populations.

## Materials and methods

### Cohort from which vaginal *Lactobacillus* strains were isolated

Vaginal *Lactobacillus* isolates were derived from women (16–22 year old) enrolled in the Women’s Initiative in Sexual Health (WISH) study [68], approved by the Human Research Ethics Committees of the University of Cape Town (UCT HREC #267/2013). Women who were ≥18 years old provided written informed consent and those <18 years provided written assent, and consent was obtained from their parent(s) or legal guardian(s). Eligibility criteria included being HIV negative, generally healthy, not pregnant or menstruating at the time of sampling. Participants were tested for BV (Nugent scoring) and STIs (including *C*. *trachomatis*, *N*. *gonorrhoeae*, *T*. *vaginalis*, *Mycoplasma genitalium*, HSV-2, *Hemophilus ducreyi*, *Treponema pallidum* and *Lymphogranuloma venerum*). The vaginal microbiota was characterised by 16s rRNA gene sequencing [36]. Cervicovaginal secretions were collected using menstrual cups, diluted 1:4 in PBS and stored with glycerol (20%v/v) at -80°C for isolation of *Lactobacillus* strains.

### Probiotics from which commercial *Lactobacillus* strains were isolated

Commercially available probiotics included Reuterina Femme (oral capsules, Ascendis Pharma, South Africa), Provacare Probiotic Vaginal Care (vaginal capsules, Provacare, Canada), Vagiforte Plus (oral capsules and vaginal tablet, Bioflora CC, South Africa), Vagiforte Plus Combo Pack (oral capsules and vaginal spray, Bioflora CC, South Africa), Gynophilus (vaginal pessary, biose, France) and Muvagyn (vaginal pessary, Hälsa Pharma GmbH, Germany).

### Isolation of *Lactobacillus* strains

Cervicovaginal secretions or commercial probiotics dissolved in De-Man-Rogosa-Sharpe (MRS) were streaked onto MRS agar plates and incubated anaerobically (37°C, 48 hours). Single colonies with distinct morphologies were picked and re-streaked until homogenous. Pure colonies were inoculated into MRS broth, grown anaerobically (37°C, 48 hours), and stocks stored with glycerol (20% v/v) at –80°C. Matrix Assisted Laser Desorption/Ionization time-of-flight (MALDI-TOF, MALDI Biotyper, Bruker Daltonik, USA) was used to identify *Lactobacillus* spp. To determine bacterial concentrations at a standardized optical density (OD), overnight cultures were adjusted to OD_600nm_ 0.1±0.01, serial dilutions plated onto MRS agar plates that were incubated anaerobically (37°C, 48 hours), colonies were counted, and CFU/mL calculated (S2 Fig).

### Confirmation of *Lactobacillus* species identities by 16S rRNA gene sequencing

To confirm MALDI-TOF species identification, single colonies were incubated (37°C for 20 minutes, followed by 90°C for 15 minutes) in Tris-EDTA buffer (pH 7.4) with 20ng/mL proteinase K (BioLabs, USA). PCR reactions contained 8% (v/v) template, 0.5uM of F27 and R5 primers [69], 0.2mM PCR nucleotide mix (Promega, USA), 1x GoTaq Flexi Buffer (Promega, USA), 1.25 units GoTaq G2 Flexi DNA polymerase (Promega, USA), 2.5mM MgCl2 (Promega, USA) and nuclease-free H_2_O. PCR conditions were 96°C for 5 minutes; 30 cycles at 94°C for 30 seconds, 55°C for 30 seconds and 72°C for 1.5 minutes, and a final elongation at 72°C for 7 minutes. Products were Sanger sequenced at Macrogen Europe. 16S rRNA sequences were aligned to National Centre for Biotechnology Information (NCBI) database sequences with the Basic Local Alignment Search Tool (BLAST) algorithm, selecting the highest percentage identity match over 99% as the species identity.

### Ability of *Lactobacillus* isolates to grow at low pH conditions

Cultures standardized to OD_600nm_ 0.1±0.01 in MRS broth adjusted to pH 3.5, 4.0, 4.5 or 6.0 using HCl were incubated in triplicate under anaerobic conditions in 96-well plates at 37°C for 48 hours. OD_600nm_ was measured at 0, 3, 6, 12, 18, 24, 36 and 48 hours and area under the curve (AUC) was calculated for each isolate. Experiments were repeated three times.

### Ability of *Lactobacillus* isolates to lower culture pH

Cultures adjusted to OD_600nm_ 0.1±0.01 in MRS broth were grown anaerobically at 37°C, and culture supernatant pH measured at 3, 6, 12, 24 and 48 hours using a calibrated pH meter. Experiments were performed in duplicate.

### Measurement of anaerobic lactate and H_2_O_2_ production

Cultures adjusted to OD_600nm_ 0.1±0.01 were incubated anaerobically for 24 hours at 37°C. Supernatants were filtered (0.2 μM) and concentrations of D- and L-lactate measured using colorimetric assays (Sigma-Aldrich, USA). H_2_O_2_ concentrations were measured using a fluorometric kit (Sigma-Aldrich, USA), according to the manufacturer’s instructions. Experiments were performed in duplicate.

### Antimicrobial activity

*G*. *vaginalis* (ATCC 14018 and five vaginal isolates) and *P*. *bivia* (ATCC 29303 and five vaginal isolates) were grown in brain heart infusion (BHI) supplemented with 5% horse blood, standardised to OD_600nm_ 0.4±0.01, and 100 μL was added in triplicate to 96-well plates. Abiotic *Lactobacillus*-conditioned MRS (100 μL; same as used for lactate measurement) was added and incubated anaerobically at 37°C for 48 hours. OD_600nm_ was measured at 0, 3, 6, 12, 18, 24, 36 and 48 hours. As control, growth of *G*. *vaginalis* and *P*. *bivia* strains in the presence of plain MRS, at the same ratio as abiotic culture supernatants, was measured. AUC for each growth curve was calculated and percentage of growth inhibition in the presence of *Lactobacillus*-conditioned MRS compared to the control calculated. Experiments were repeated three times.

### Adhesion to human ectocervical epithelial cells

Ca Ski cells (ATCC CRL-1550) were grown to 80% confluence in 10% fetal calf serum (FCS) Dulbecco’s Modified Eagle Medium (DMEM) in 24-well cell culture plates (37°C, 5%CO_2_). *Lactobacillus* isolates (4.2x10^6^ CFUs) were added in triplicate and co-cultured for 3 hours at 37°C (5%CO_2_), after which unbound bacteria were gently removed by washing the cells three times with PBS. Cells and bound bacteria were detached using 0.1% Triton X-100, serially diluted, plated onto MRS agar plates, and incubated anaerobically at 37°C for 48 hours. The percentage of adhered bacteria was calculated as the number of bound cells compared to total number of bacterial cells initially added. Experiments were repeated three times.

### Impact on cervical cell viability and cytokine responses

Ca Ski cells were grown in 10% FCS DMEM to 80% confluence at 37°C in 5% CO_2_. *Lactobacillus* strains (4.2x10^6^ CFUs) were added in duplicate and incubated for 24 hours (37°C, 5% CO_2_). Ca Ski cells incubated without *Lactobacillus* strains were included as control. Supernatants were harvested and filtered (0.22 μm, Corning Costar Spin-X, Sigma-Aldrich, USA) to exclude bacterial cells. A Bio-Plex Pro Human Cytokine 27-Plex Luminex kit (Lot 64064139, Bio-Rad, USA) was used to measure concentrations of cytokines, chemokines and growth factors in the culture supernatant, according to the manufacturer’s instructions. Data were acquired using a Bio-Plex Suspension Array Reader (Bio-Rad Laboratories Inc, USA), and a 5PL regression line was used to determine concentrations from standard curves. All values below the detection limit were recorded as half of the lowest measured concentration for each cytokine. Viability of Ca Ski cells after co-culture was evaluated using trypan blue staining (0.4%, Sigma-Aldrich, USA).

### Antibiotic susceptibility testing

Double-layer disc diffusion was used to determine the susceptibility to metronidazole (5μg), clindamycin (2μg), penicillin (2μg) and amoxicillin (10μg; Davies Diagnostics, South Africa), as described previously [58]. These experiments were performed in duplicate. For rifampicin and rifabutin (Sigma-Aldrich, USA), minimal inhibitory concentrations (MICs) were determined using two-fold serial dilutions according to European Committee on Antimicrobial Susceptibility Testing (EUCAST) 2019 guidelines, with concentrations ranging from 5–0.00488μg/mL for rifabutin and 25–0.024μg/mL for rifampicin. MICs below the lowest or above the highest concentration tested were assigned a MIC half of the lowest or twice the highest concentration tested, respectively. Experiments were performed in duplicate. For a subset of *Lactobacillus* strains (n = 20), broader antibiotic susceptibility profiles were determined using Sensititre GPALL1F plates (including ampicillin, cefoxitin, chloramphenicol, ciprofloxacin, clindamycin, daptomycin, erythromycin, gentamicin, levofloxacin, linezolid, moxifloxacin, nitrofurantoin, oxacillin, penicillin, quinupristin/dalfopristin, rifampin, streptomycin, tetracycline, tigecycline, trimethoprim/sulamethoxazole and vancomycin; Thermo Fisher Scientific Inc., USA), according to the manufacturer’s instructions.

### Whole genome sequencing

Genomic DNA was extracted from *Lactobacillus* isolates using the Quick-DNA Miniprep Kit (Zymo Research, USA) following the manufacturer’s instructions after a pre-lysis step which consisted of resuspension of the cell pellet in 200 μl PrimeStore Molecular Transport Medium (Longhorn Vaccines-Diagnostics, USA) and storage at -80°C for 24 hours. Libraries for sequencing were prepared using the Nextera DNA Flex kit (Illumina, USA), according to the manufacturer’s instructions, and sequenced using the Illumina MiSeq Reagent Micro Kit v2 on an Illumina MiSeq instrument in a paired-end, dual indexed 2 x 151 cycle sequencing run. Draft *de novo* assemblies were prepared using a customised assembly pipeline. Briefly, raw reads were trimmed using Trimmomatic v0.3.9 [70] using a sliding window approach (Phred quality cut-off of 15 averaged across 4 bases), a leading/trailing quality cut-off of 3 and the removal of reads below 30bp. Trimmed reads were assembled using SPAdes v3.13.0 [71] using the ‘—careful’ flag and omitting the initial read error correction step. To improve the draft assemblies, gap closure and contig extension were performed using GMcloser v1.6.2 [72]. The quality of the final assemblies for each strain was assessed using Quast v5.0.2 [73]. Rapid Annotation of microbial genomes using Subsystems Technology (RAST) was used to annotate the de novo assemblies [74].

### Identification of putative antimicrobial resistance (AMR) determinants, prophages and mobile elements

Annotated draft assemblies were screened for the presence of putative antimicrobial resistance determinants using Resfinder v3.2. [75], CARD [76] and AMRFinder [77]. Putative prophages were identified using the online PHASTER server [78] [https://phaster.ca/]. CRISPR-Cas systems were identified using the CRISPRCasFinder online tool [79] [https://crisprcas.i2bc.paris-saclay.fr/] and spacer targets were identified using CRISPRTarget [80].

### Statistical analyses

GraphPad Prism6 (GraphPad Software, USA), STATA version 11.0 (StataCorp, USA) and RStudio were used to generate graphs and analyse data. The Mann-Whitney U test was used for non-parametric variables, and Spearman Rank test was used for non-parametric correlations. The Kruskal-Wallis one-way analysis of variance was used to compare variables with three or more groups. A false-discovery rate step-down procedure [81] was used to adjust p-values for multiple comparisons, and adjusted p<0.05 were considered significant.

To rank *Lactobacillus* strains according to PPP criteria, a scoring system was developed. For each characteristic, *Lactobacillus* isolates were ranked into quartiles relative to the whole group: Scored “0” for characteristics <25^th^ percentile (relative to all isolates); “1” for characteristics between 25–50^th^ percentile; “2” for characteristics between 50–75^th^ percentile, and “3” for characteristics between >75^th^ percentile. Because some characteristics may correlate (e.g. ability to lower pH and H_2_O_2_ production), all scores were weighted to account for co-linearity: Weight = 1-(sum of column Spearman Rho÷sum of column score).

## Supporting information

S1 Fig Temporal comparison of the ability of vaginal and probiotic *Lactobacillus* isolates to lower culture pH. pH of vaginal *L*. *crispatus* (A), *L*. *gasseri* (B), *L*. *vaginalis* (C), *L*. *jensenii* (D), *L*. *mucosae* (E) and the probiotic (F) cultures were measured over 48 hours. Probiotics include *L*. *reuteri*, *L*. *rhamnsus* and *L*. *acidophilus* strains. As control, the pH of abiotic MRS was measured (shown in light grey). ATCC reference strains are shown as black lines. Physiological vaginal pH of women with optimal microbiota is shown as grey shading.
(TIFF)

S2 Fig **Comparison of vaginal *Lactobacillus* spp. concentrations** (measured in CFUs) at a standardized OD_600nm_ 0.1 (±0.01): *L*. *crispatus* (A), *L*. *gasseri* (B), *L*. *vaginalis* (C), *L*. *jensenii* (D), and *L*. *mucosae* (E). Isolates from BV/STI-negative women are shown by plain bars, those from BV and/or STI-positive women are patterned and ATCC strains are shown by black bars.
(TIFF)

S1 Table Assembly statistics of WGS. The table shows the assembly statistics for the sequenced vaginal *Lactobacillus* strains. The sequences have been deposited in the European Nucleotide Archive (ENA) under accession number PRJEB37955.
(DOCX)
