# Supplementary figures and images for: Exploring potential of vaginal Lactobacillus isolates from South African women for enhancing treatment for bacterial vaginosis
Source: PLoS Pathog. 2020 Jun 4;16(6):e1008559. doi: 10.1371/journal.ppat.1008559 (PMC7271994; doi:10.1371/journal.ppat.1008559)

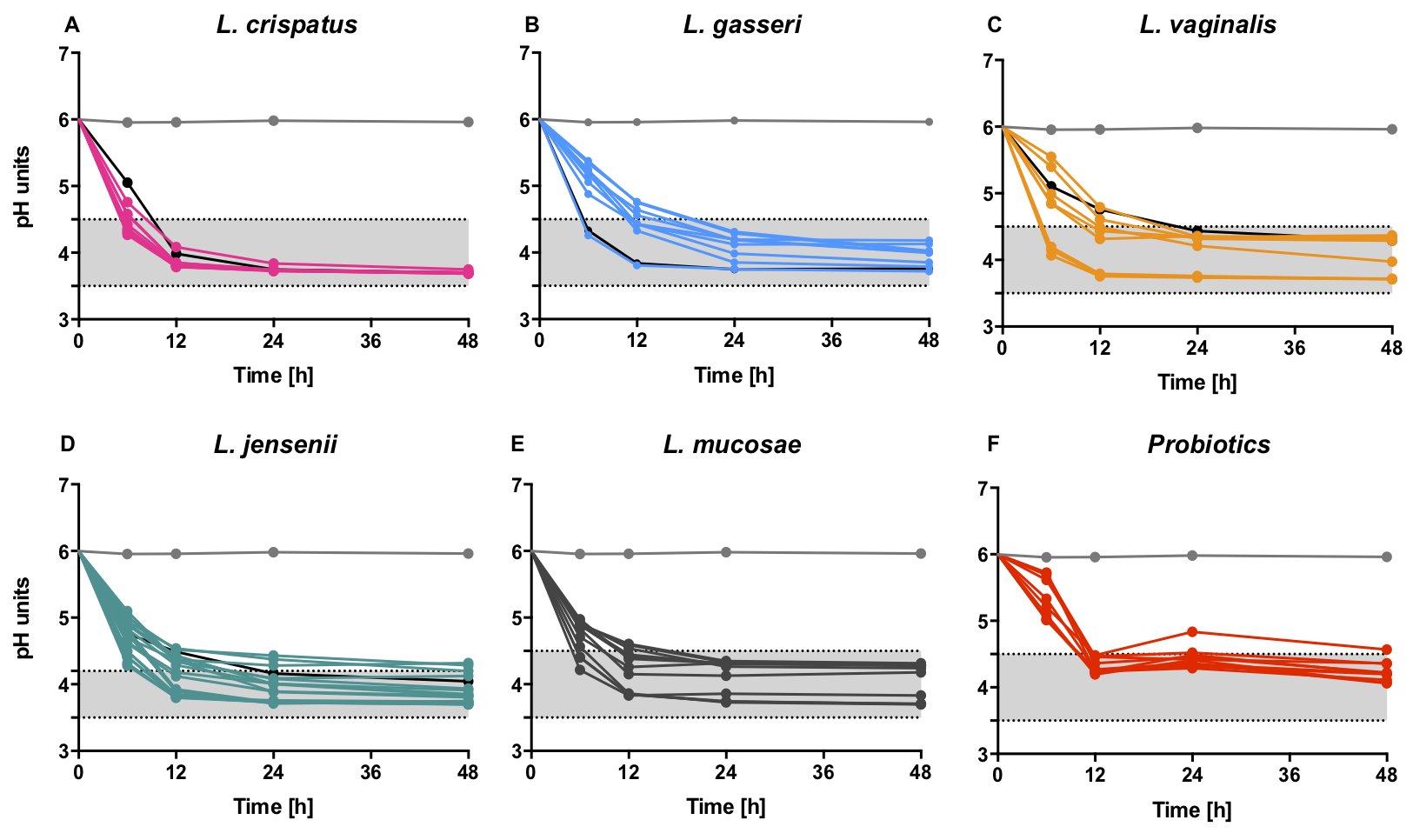

Supplement: S1 Fig — pH of vaginal L. crispatus (A), L. gasseri (B), L. vaginalis (C), L. jensenii (D), L. mucosae (E) and the probiotic (F) cultures were measured over 48 hours. Probiotics include L. reuteri, L. rhamnsus and L. acidophilus strains. As control, the pH of abiotic MRS was measured (shown in light grey). ATCC reference strains are shown as black lines. Physiological vaginal pH of women with optimal microbiota is shown as grey shading. (TIFF) [file ppat.1008559.s001.tiff]

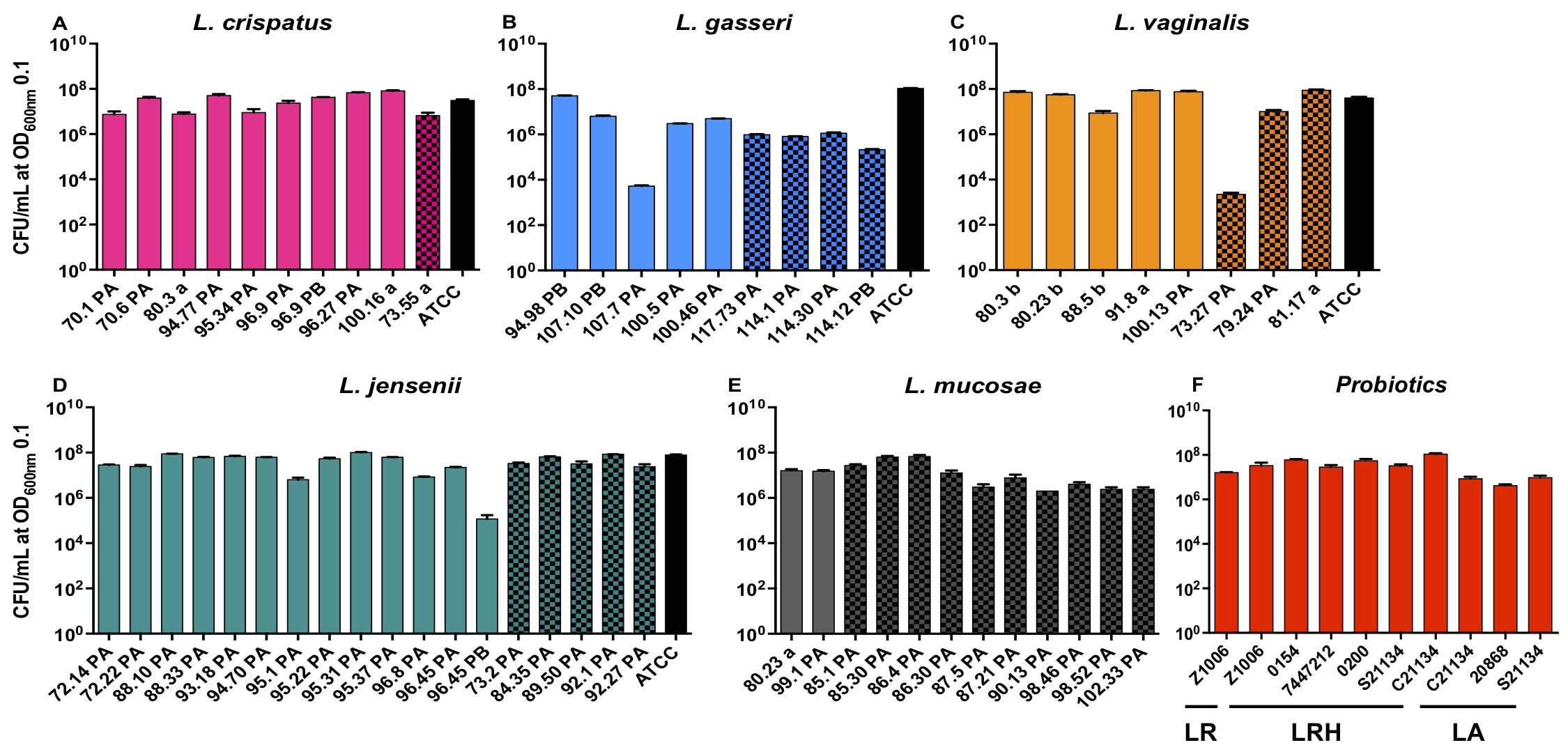

Supplement: S2 Fig — Comparison of vaginal Lactobacillus spp. concentrations (measured in CFUs) at a standardized OD600nm 0.1 (±0.01): L. crispatus (A), L. gasseri (B), L. vaginalis (C), L. jensenii (D), and L. mucosae (E). Isolates from BV/STI-negative women are shown by plain bars, those from BV and/or STI-positive women are patterned and ATCC strains are shown by black bars. (TIFF) [file ppat.1008559.s002.tiff]
