# Supplementary material for: Exploring potential of vaginal Lactobacillus isolates from South African women for enhancing treatment for bacterial vaginosis
Source: PLoS Pathog. 2020 Jun 4;16(6):e1008559. doi: 10.1371/journal.ppat.1008559 (PMC7271994; doi:10.1371/journal.ppat.1008559)
Supplement: S1 Table — The table shows the assembly statistics for the sequenced vaginal Lactobacillus strains. The sequences have been deposited in the European Nucleotide Archive (ENA) under accession number PRJEB37955. (DOCX) [file ppat.1008559.s003.docx]

**S1 Table. Assembly statistics of WGS.**

|  | **Vaginal *Lactobacillus* strains** | | | | |
| --- | --- | --- | --- | --- | --- |
| **Metric** | **70.6 PA** | **73.55 a** | **90.13 PA** | **94.98 PB** | **95.1 PA** |
| **Genome size (Bp)** | 2268450 | 2191574 | 1997735 | 1924077 | 1626426 |
| **GC (%)** | 36.78 | 36.67 | 46.63 | 34.9 | 34.4 |
| **N CDS** | 2570 | 2426 | 2085 | 1927 | 1556 |
| **Total CDS length (Bp)** | 2012370 | 1940628 | 1742481 | 1723275 | 1474692 |
| **Coding (% of genome)** | 88.71 | 88.55 | 87.22 | 89.56 | 90.67 |
| **Coding density (CDS/Mbp)** | 1132.93 | 1106.97 | 1043.68 | 1001.52 | 956.70 |
| **N tRNA** | 61 | 61 | 76 | 55 | 54 |
| **N rRNA** | 4 | 3 | 4 | 4 | 3 |
| **N Contigs** | 268 | 81 | 57 | 109 | 65 |
| **N50** | 21952 | 108270 | 112131 | 41219 | 71941 |
| **L50** | 33 | 7 | 7 | 14 | 6 |
| **Coverage (Theoretical X)** | 106.76 | 102.71 | 117.96 | 125.54 | 142.56 |
